# Inverse shielding and mutual exclusion for PET-MR hybrid imaging concerning induced positronium hyperfine splits radiations

**DOI:** 10.1038/s41598-023-44303-3

**Published:** 2023-11-22

**Authors:** Kelin Wang, M. Saiful Huq

**Affiliations:** 1 UPMC Hillman Cancer Center, Pittsburgh, PA USA; 2 University of Pittsburgh School of Medicine, Pittsburgh, PA USA

**Keywords:** Cancer imaging, Positron-emission tomography, Imaging techniques

## Abstract

Prevalent PET imaging reconstructs 2γ-photon pairs emitted after an annihilation from para-positronium (p-Ps) and rejects 3γ events from ortho-positronium (o-Ps) as noises. The 3γ/2γ decay ratio is ~ 3/7 in human body theoretically but in fact significantly lower due to *pick-off process*, hence PET imaging quality is well controlled. In a PET-MR hybrid unit, the MR magnetic field alters positronium decay patterns through *magnetic quenching*: all o-Ps and excited p-Ps states are split into finer quantum states under strong magnetic field, thus transitions between some triplet and singlet finer states (m_z_ = 0) were no longer forbidden, thus some o-Ps converts to p-Ps spontaneously by emitting hyperfine split (HFS) photons, which also drops 3γ/2γ ratio hence helps PET imaging quality. However, *inverse magnetic quenching* might also occur if any external source of HFS frequencies is nearby, thus many p-Ps convert to o-Ps by absorbing those HFS photons (induced HFS transitions). This will dramatically increase 3γ/2γ ratio and hence degrade PET imaging quality instantaneously. The HFS spectrum lies in a broad range of microwaves, from 0.02 to 200 GHz. To prevent inverse magnetic quenching, it is necessary to block external microwave sources outside the hybrid vault, by adding a thin metal layer at all directions of the vault. This could be achieved by adopting the metallic *Faraday Cage,* which was originally for MR shielding, with possible amendment if necessary. The frequencies of excitation pulses in MR imaging overlap with HFS spectrum, however, the chance for mutual interference during hybrid imaging is small, hence there seems no need to veto each other during hybrid scans.

## Introduction

Following the advancement of PET-CT hybrid scanner at the beginning of new millennium^1,2^, the PET-MR hybrid unit was developed about a decade later^3,4^. Hybrid medical imaging that combines two different modalities into one integrated unit has many advantages in diagnoses: compensated information, simultaneous scanning, reciprocal supports in reconstruction, and automatic internal co-registration, etc. Despite all the advantages, the drawback of hybrid imaging has been the interference between 2 different imaging modalities during scanning, because each imaging modality might require very specific imaging conditions. In a PET-CT hybrid unit, the scattered photons generated in PET imaging cause more noises in CT, thus reconstruction algorithms with advantage on noise filtering, e.g., filter back-projection (FBP), are favored with respect to other algorithms^5^. As of now, major concern on PET-MR interference in hybrid scanning has focused on compatibility of MR and PET electronics in data acquisition^6–10^. Other concerns included attenuation correction for PET while the CT (in the PET/CT unit) was replaced by a MR^11^, and minor influence on PET spatial resolution due to the circular motion of positronia under magnetic field^12–15^. Another important concern on PET-MR interference, probably more essential but never be proposed hence much less addressed, is positronium hyperfine split (HPS) radiations under MR magnetic field, and the correlated impact on PET imaging quality. In this study, we attempted to address this concern in a broad scope of positronium triplet/singlet composition, quenching principles, induced HPS excitations for PET imaging quality, and inverse shielding against external sources to induce HPS excitations.

## Clinical PET imaging quality due to 3γ/2γ annihilation ratios

Since being invented, clinical PET imaging only attempted to reconstruct 2γ photon pairs from annihilations, relinquishing all 3γ events as noises due to multiple reasons. Hence, scrutinizing 3γ/2γ events ratio is crucial for PET imaging quality. Depending on electron densities in matters, positrons could directly annihilate with electrons in large, e.g., in metals, or partially go through the intermediate process of positronium (Ps) decay^16–18^. In biological environment such as mammals, the chance for direct annihilations is ~ 60%, primary into 2γ photons, and for Ps decay is ~ 40%^19^. Theoretically, a Ps has 1/4 chance in para-spinal state (p-Ps, singlet, decaying to even number of γ-photons, primarily 2γ) and 3/4 chance in ortho-spinal state (o-Ps, triplet, decaying to odd number of γ-photons, primarily 3γ), without considering the interferences by other factors, e.g., magnetic fields, collisions etc. For any given Ps, annihilations to 4γ and 5γ events are very rare: the branching ratios of 4γ/2γ and 5γ/3γ are both ~ 10^–6^, and even smaller for more photon decays^20^. The chances for other type of decays are also negligible, e.g., an o-Ps could “vanish” by decaying to an undetectable neutrino-antineutrino pair ($v\overline{v }$) with ~ 10^–11^ probability^21^. Counting the direct annihilations, the entire probability for 2γ annihilation events in biological environment should be ~ 70%, and that for 3γ events is ~ 30%, hence the 3γ/2γ ratio is ~ 3/7 in theory.

In clinical PET imaging situations, the 3γ/2γ ratio could be much less than 3/7 due to multiple factors. Studying the energy profile for PET imaging may reveal important information of 3γ/2γ ratio. A typical PET profile with zero threshold consists of 3 peaks regardless of scintillating detector types: a sharp peak at ~ 511keV, and 2 smaller but broader peaks of similar amplitudes at ~ 341keV and ~ 195keV, respectively. As known, γ-photons interact with scintillator detectors by either photoelectric (PE) effect or Compton scattering. In either process a recoiled electron is generated, escaping from scintillator binding ~ 200eV or 0.2keV. ($E\approx {-Z}_{eff}^{2}/13.6{n}^{2}$ where *Z*_*eff*_ ≈73 for BGO scintillator^22^,* n* = 1,2 for outer shell electrons). Comparing to the 511keV, this amount of binding energy normally was ignored in the profile. Those 3 peaks in a PET energy profile are the kinetic energies of different groups of recoiled electrons, overlapping for both 2γ and 3γ events.

### The 511keV, 341keV peaks: PE and Compton edges for 2γ annihilations

When a *γ*-photon from a *2γ* annihilation goes through a PE process in the scintillator, a bound electron absorbs the entire 511keV and escapes. Stopping the energetic recoiled electron, a scintillator generates multiple visible photons, which will be either amplified by a photomultiplier tube (PMT) or directly read by an avalanche photodiode (APD)^23^. Since 2γ events are predominated, those PE events build up a sharp peak of ~ 510.8keV sharp in profile.

In a Compton scattering, a recoiled electron carries some energy of the incident γ-photon. Assuming *m*_*e*_ is electron/positron mass and *θ* is the scattered angle, the incident energy is *m*_*e*_*c*^*2*^ for each of the 2γ annihilations, and the energy of the scattered γ-photon *Eʹ* is simplified to:

1 $${E}{\prime}=\frac{{m}_{e}{c}^{2}}{2-cos\theta }$$

When an incident *γ*-photon is back-scattered, *θ* = 180°, thus *E*’ = *m*_e_*c*^2^/3. The energy transferred to the recoiled electron by scattering is 2*m*_e_*c*^2^/3 = 340.7keV. This is known as the “*Compton Edge*”^24^, or more strictly, the *2γ* (annihilation) *Compton Edge.* If considering the ~ 200eV binding energy, the energy peak for *2γ Compton Edge* is ~ 340.5keV.

### Interpretation for 195keV broad peak and re-evaluation of 340.5keV peak

Properties of 3γ events in PET imaging were never earnestly studied, mainly because prevalent PET scanners have very low collecting efficiency even for 2γ reconstructions^25^, hence improving detector sensitivity for 2γ events seemed more imperative than overcoming the difficulties of triggering 3γ coincidences in a PET scanner. For this reason, the broad peak at ~ 195keV at PET energy profile was never interpreted legitimately.

The geometry of 3*γ* separation from o-Ps decay has been controversial. According to Ore and Powell (1949), the geometry of separation by 120° each in a o-Ps decay is not very significant, instead, there should be a broad distribution for all geometries^26^. For 120°-separation geometry, each *γ*-photon should have equal energy 2m_e_c^2^/3 = 340.7keV, hence a PE broad peak of 340.5keV should be formed considering the ~ 200eV electron binding energy. However, 340.5keV is also the *2γ Compton Edge*, thus the *3γ* PE peak overlaps with the *2γ Compton Edge.* That is, strictly saying, the 340.5keV peak in the PET energy profile is a *composite* peak of the *2γ Compton Edge* and the 3γ PE peak for those equal-energy 3γ o-Ps decay.

If the assumption above is correct, a *Compton Edge* for 3γ annihilations with *γ*-photon energy 340.7keV should be also seen in the PET energy profile. We here re-write the energy *E’* for the scattered *γ*-photon in a *Compton* scattering, considering the incident *γ*-photon has an energy of 2*m*_*e*_*c*^*2*^/3:

2 $${E}{\prime}=\frac{2{m}_{e}{c}^{2}}{5-2cos\theta }$$

For backscattering *θ* = 180°, thus the scattered photon has energy *E’* = 2m_e_c^2^/7. Subtracting this amount of energy from original incident energy 340.7keV (or 2m_e_c^2^/3), we have the energy for recoiled electron is: 8m_e_c^2^/21 = 194.7keV. Considering the ~ 200eV binding energy of electron, the exact peak of *3γ Compton Edge* should locate at ~ 194.5keV, which exactly fits the 3^rd^ broad peak in PET energy profile. However, Ore and Powell’s calculations seemed also reasonable, because the 194.5keV peak is broad.

## Clinical “pick-off quenching” to by-pass forbidden rule in PET imaging

As known, an o-Ps (triplet Ps) always has slightly higher energy level than a p-Ps (singlet Ps) for any given principal quantum number *n* (*n* ≥ *1*). This energy difference between a triplet and a singlet of the same quantum number *n* is known as hyperfine split (HFS) of positronium. For instance, the HFS at ground state (between 1^1^S_0_ and 1^3^S_1_) is 8.425 × 10^–4^ eV, equivalent to 203.4GHz microwave, 2 orders lower than thermal energy photons ~ 0.025eV; and at first excited state (between 2^1^S_0_ and 2^3^S_1_) the HFS is 25.4GHz, 3 orders lower than thermal energy^27^. However, an o-Ps cannot convert to a p-Ps by emitting an HFS photon spontaneously, because in an o-Ps (e.g., 1^3^S_1_ triplet state) the ${e}^{+}{e}^{-}$ spin parallelly to each other (↑↑ or ↓↓) whereas in a p-Ps (e.g., 1^1^S_0_ singlet state) they spin antiparallelly (↓↑ or ↑↓). That is, transitions between triplet and singlet states are *forbidden* because it is impossible to flip spin directions. Recent experiments to verify QED and to discover new physics beyond Standard Model had validated this prediction—the chance for 1^3^S_1_ → 1^1^S_0_ transition (or vice versa) is only ~ 10^–8^, very close to zero^28^. The incredulous tiny amount 10^–8^ is caused by quantum tunneling effect.

**Figure 1:**
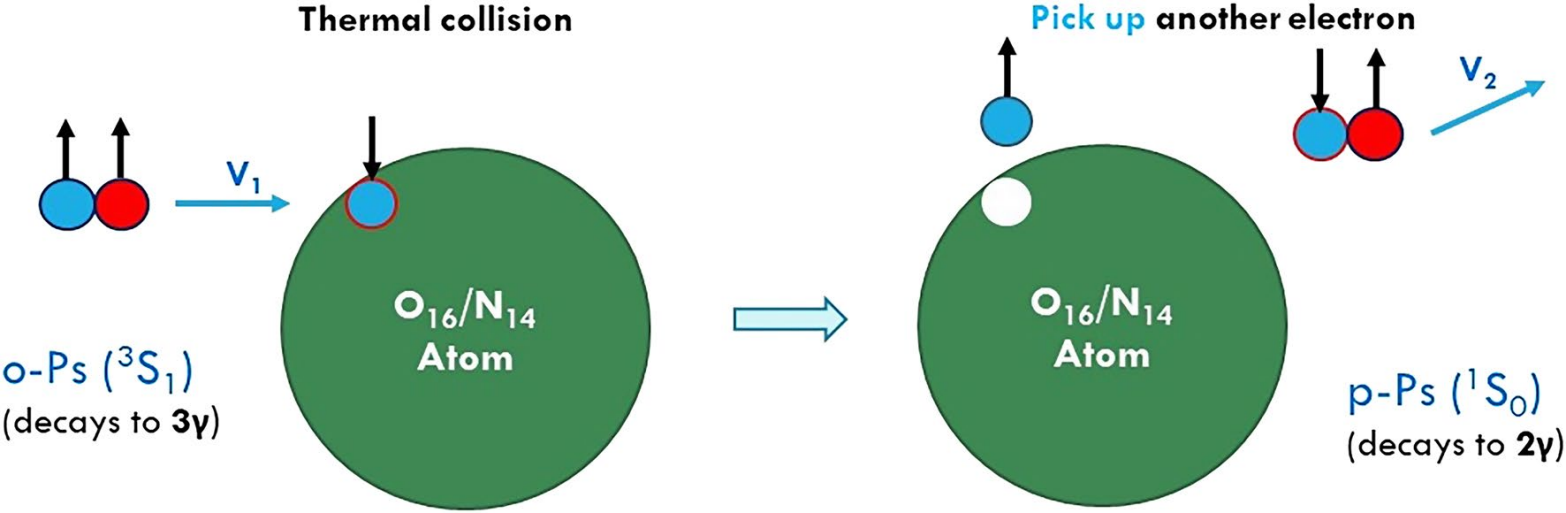
Pick-off quenching.

At body temperature all Ps are in thermal motion with kinetic energy *k*_*B*_*T* (*k*_*B*_ is *Boltzmann Constant* and *T* is the absolute temperature). Considering the very short free path in tissues, the thermal Ps collide with surrounding molecule frequently. The average lifetime for p-Ps is 0.124ns and that for o-Ps is 142ns, about 1100 times longer. That is, an o-Ps collides over 1000 more times than a p-Ps before annihilation. In some of those collisions, an o-Ps (e.g., ↑↑) *picks up* a binding electron with antiparallel spin (↓) from the molecule, meanwhile *drops off* the original electron (spin ↑) in the Ps, and then forms a new Ps in which electron and positron have opposite spin (↓↑). Obviously, the newly formed Ps is an p-Ps (Fig. 1). That is, through this special “pick-off” collision process, an o-Ps converts into a p-Ps. In clinical imaging environment, the *pick-off* process occurs frequently, thus the total number of o-Ps might drop significantly depending on tissue types. This phenomenon of reducing o-Ps numbers is known as “pick-off quenching” of triplets, which could be verified by comparing the mean lifetime of o-Ps in human body^29^. A p-Ps could also convert to an o-Ps through a similar *inverse-pick-off* process, picking an electron with parallel spin. However, considering the much less collisions for p-Ps than o-Ps, the chance for p-Ps → o-Ps through *inverse-pick-off* process is about 3 orders smaller. In general, the “pick-off quenching” of o-Ps is the very reason that 2γ events predominated in typical PET imaging. Obviously, “pick-off quenching” enhances PET imaging quality by dropping 3γ/2γ ratio.

### Prevalent consideration on 3γ/2γ ratio in clinical PET imaging

Since the *pick-off quenching* theory was proposed, an estimation used to be well accepted that over 99% of annihilations result in 2γ events in human body during PET imaging^30^. However, a recent measurement, not in clinical situation though, revealed that the *pick-off* process only contributes up to 6% of triplet quenching^31^. As of now, there has been no published evidence on precise 2γ/3γ ratio considering the *pick-off quenching* effect. In clinical PET imaging, there *might* be a good chance that up to ~ 20% 3γ events depending on tissue densities.

### Implementation of pick-off process: positron annihilation spectroscopy (PAS)

Positron annihilation spectroscopy (PAS), sometime also referred to positron annihilation lifetime spectroscopy (PALS), was developed since 1970s^32,33^, and become well recognized in the new millennium^34–37^. When positrons enter materials, direct annihilations typically occur within ~ 10ps in the electron-rich environment, whereas positronia decay much later if any voids are present in the materials. Analyzing time information of annihilations may indicate the voids in materials, which was the original idea for PAS. Since o-Ps could convert to p-Ps through pick-off effect hence annihilate sooner, and the chance of pick-off effect is highly environmental dependent, measuring o-Ps becomes a very sensitive indicator for mesoporous detection in tissues and materials in microscopic scales^34–37^.

## Magnetic quenching to overcome forbidden rule

### Magnetic quenching to convert o-Ps to p-Ps

Other ways to flip a spin direction in a Ps, hence converting an o-Ps to a p-Ps or vice versa, are to apply an external magnetic field (Zeeman Effect) and/or electric field (Stock Effect). For instance, when a MR magnetic field (e.g., ≥ 1.0T) is applied, the resultant spin of a Ps (either o-Ps or p-Ps) aligns with the strong magnetic field. The energy difference ΔE between adjacent split states can be written as:

3 $$\Delta E={m}_{z}{\mu }_{B}B$$

where *m*_*z*_ is quantum angular momentum, *µ*_*B*_ is the Bohr magneton, and *B* is magnetic field. As results, the degenerate energy levels of an o-Ps and excited states of a p-Ps (2^1^S_0_ or above) will split into new fine quantum states, hence energy transitions between some o-Ps and p-Ps between certain split states (m_z_ = 0, assuming *z-*direction is the magnetic field) are allowed.

**Figure 2:**
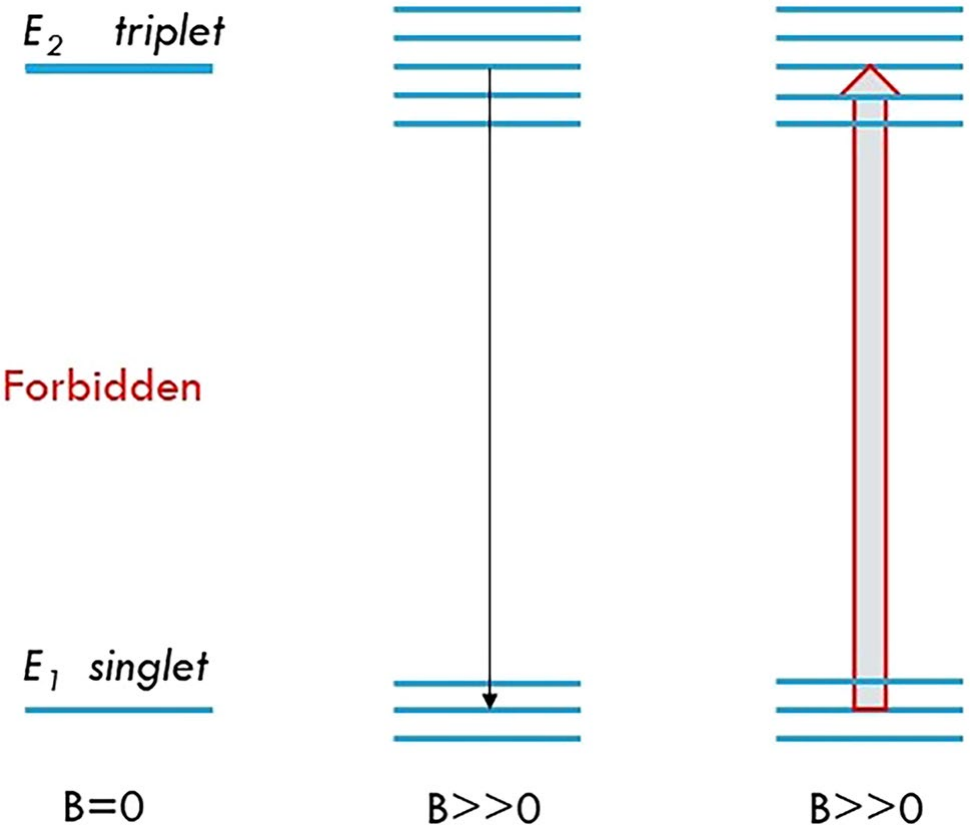
Induced HFS excitations and Inverse *Magnetic Quenching.* Left panel: transitions are forbidden between triplet and singlet if there is no magnetic field. Central panel: spontaneous transition between split triplet m_z_ = 0 and split singlet m_z_ = 0 under strong magnetic field B. Right panel: induced transitions from split singlet (m_z_ = 0) to split triplet (m_z_ = 0) under strong magnetic field if a microwave source of HFS frequency is available.

Since the energy levels of the split o-Ps are higher than that of p-Ps, the o-Ps in split m_z_ = 0 state may spontaneously fall to p-Ps m_z_ = 0 state, by emitting an HPS photon (Fig. 2, central panel).

Consequently, a 2γ annihilation rather than 3γ ultimately occurs. This phenomenon that triplet-rich positronia convert to more singlets under a strong magnetic field is known as “magnetic quenching”^38,39^. For instance, a 2^3^S_1_ (o-Ps) falls to 2^1^P_1_ (p-Ps) by emitting a photon of 11.2GHz photon^39^, hence a 2γ annihilation occurs by 2^1^P_1_ rather than a 3γ by 2^3^S_1_. Obviously, magnetic quenching also improves PET imaging quality by dropping 3γ/2γ ratio. Magnetic quenching can be observed with PALS^40^.

### Contrast resolution improvement of PET by Magnetic quenching

In any given voxel of 3-dimensional PET image, the contrast resolution, representing by significance of signal to noise *S*, can be defined as:

4 $$S=\frac{{N}_{2\gamma }}{\sqrt{{N}_{2\gamma Comp}+{N}_{3\gamma }+{N}_{Other}}}$$

*N*_*2γ*_ is the signal events for 2γ coincidences formed by *Photoelectric effect* after the pick-off process, which include both direct 2γ annihilation and p-Ps decays. *N*_*2γComp*_ is the *Compton scattering* events of 2γ coincidences, the major noises. *N*_*3γ*_ is the 3γ events caused by o-Ps, which were considered as noise due to lower energies. *N*_*Other*_ is all other noise events. When a strong external magnetic field is applied, most of o-Ps will convert to p-Ps shortly, which means increasing the 2γ (signal) events, at the meanwhile, decrease the 2γ (noise) events of equal number. Denoting this number of events as *ΔN*, the new 2γ event *N’*_*2γ*_ = *N*_*2γ*_ + *ΔN*; and the 3γ events decreases by the same number *N’*_*3γ*_ = *N*_*3γ*_ – *ΔN*, hence *S* is changed to S’:

5 $${S}{\prime}=\frac{{{N}{\prime}}_{2\gamma }}{\sqrt{{N}_{2\gamma Comp}+{{N}{\prime}}_{3\gamma }+{N}_{Other}}}=\frac{{N}_{2\gamma }+\Delta N}{\sqrt{{N}_{2\gamma Comp}+{N}_{3\gamma }-\Delta N+{N}_{Other}}}$$

Comparing formula (4) and (5), it is easy to see that *S’* > *S*. That is, magnetic quenching improves the contrast resolution.

### Magnetic quenching nature: conducting pathways for transitions

It is important to comment that an external magnetic field (or electric field) essentially conducts some pathways between o-Ps and p-Ps, enabling energy transitions in both directions, either from o-Ps → p-Ps, or vice versa. The reason *magnetic quenching* for o-Ps occurs is than an o-Ps has higher energy level than a p-Ps, hence HPS transition happens spontaneously under magnetic field. Since an HFS photon has very tiny amount of energy, a p-Ps could possibly gain this amount of energy from environment (e.g., by collisions, etc.) and transits to an o-Ps (m_z_ = 0 state) through the same conducted pathway. This type of *inverse* transition process was also observed in singlet-rich positronia, although the chance for the inverse transitions is much smaller^40^. In typical PALS measurements, the initial positronia were mostly triplet-rich (~ 75%), hence *magnetic quenching* for o-Ps was more significant than the inverse process^39^.

For the same example above, if an external magnetic field is applied, and a microwave source of 11.2GHz (HFS frequency) is nearby, induced HFS excitations may occur if p-Ps predominate. That is, many p-Ps at 2^1^P_1_ state could jump to o-Ps 2^3^S_1_ state by absorbing those HPS photons, thus the number of p-Ps will be significantly reduced instantaneously. This theoretical phenomenon could be defined as *inverse magnetic quenching* for triplet Ps or induced magnetic quenching for singlet Ps (Fig. 2, right panel). Consequently, more 3γ rather than 2γ events will be ultimately generated in PET imaging. Since 3γ events are always treated as noises, increasing 3γ/2γ ratio through *inverse magnetic quenching* will significantly degrade PET imaging quality.

## Inverse Shielding for PET-MR Hybrid Unit

Considering the possibility of inverse magnetic quenching for triplet Ps in PET-MR hybrid imaging, shielding against external microwave sources should be implemented to a PET-MR vault to ensure PET imaging quality. In general, the purpose for vault shielding of X-ray machines, medical linear accelerators, SPECT units, CT simulators or PET-CT hybrid units are to attenuate radiations from the medical devices, hence, to protect the areas outside the vault, adjacent to vault walls, ceiling, or floor. Inverse shielding, however, is to protect the medical device (PET-MR hybrid unit, in this situation) against external radiations which could penetrate through walls or ceiling, such that the device could work properly.

For the inverse shielding of PET-MR hybrid imaging, it would be important to illustrate the entire spectrum of external sources that could induce HFS excitations.

### Populations of Ps excited states

Considering the ħ/2 spin of electron and positron, a Ps can be considered as a boson since its spin is always an integer, e.g., spin 1 at 1^3^S_1_ and spin 0 at 1^1^S_0_. The positronia inside clinical environment could be considered as Boson gas. The situation that all bosons are at the same ground states is known as Bose–Einstein condensation, which occurs only at extremely low temperature, obviously not the situation in human body. Indeed, a human body of constant temperature could be approximately treated as a *black body* with central spectrum at infrared region indicating by body temperature (~ 36.5°C), thus most Ps in human body are in excited states rather than ground states.

As shown in formula (4), the energy levels of a Ps based on Bethe–Salpeter equation^26^ and transitions between those principal quantum levels n (n ≥ 1) are listed in Table 2, without considering HFS, which are 4 orders smaller.

4 $$E=-\frac{6.8eV}{{n}^{2}}$$

For instance, the ground state (n = 1) energy is -6.8eV and the first excited state (n = 2) energy is -1.7eV, hence the transition energy between those 2 states is 5.1eV, equivalent to an ultraviolet (UV) photon of 243nm wavelength. The transition energy between n = 2 and n = 3 is 0.94eV, which is in near-infrared (NIF) spectrum. Similarly, transitions for other higher energy levels locate in middle-wavelength (MW) infrared, long-wavelength (LW) infrared and far-infrared (FIR), respectively. That is, it might not be easy for a positronium at ground state (n = 1) to be excited to the first excited state (n = 2) by directly absorbing a UV photon inside human body. However, transitions between all other energy levels (n ≥ 2) are much easier because they are all within infrared band, from NIR to FIR. Considering that infrared radiations dominate inside human body, most positronia could easily stay at different excited states (n ≥ 2) before annihilation.

### Positronium HFS spectrum

At any given principal quantum level n (n ≥ 1) of positronium, the HFS energies are about 4–5 orders lower than the energy difference between 2 adjacent principal quantum levels (n & n + 1). For instance, the transition energy from n = 1 to n = 2 is 5.1eV (Table 1), and HFS energy for n = 1 between 1^3^S_1_ and 1^1^S_0_ is 8.425 × 10^–4^ eV, equivalent to 203.4GHz (i.e., extreme high frequency (EHF) of microwave spectrum). The HFS energies (in GHz) at any given principal quantum number n (n ≥ 1) can be described as:

**Table 1:** Positronium energy levels, transitions, and transition spectrums.

Quantum number	Energy levels (eV)	Transitions	Wavelength (μm)	∆E (eV)	Spectrum
1	− 6.8	Ground state			
2	− 1.7	n = 2, n = 1	0.243	5.1	UV
3	− 0.76	n = 3, n = 1	1.31	0.94	NIR
4	− 0.43	n = 4, n = 3	3.75	0.33	MW IR
5	− 0.27	n = 5, n = 4	8.10	0.15	LW IR
6	− 0.19	n = 6, n = 5	14.9	0.083	LW IR
7	− 0.14	n = 7, n = 6	24.7	0.050	FIR
8	− 0.11	n = 8, n = 7	38.1	0.033	FIR
9	− 0.084	n = 9, n = 8	55.6	0.023	FIR
10	− 0.068	n = 10, n = 9	77.7	0.016	FIR
12	− 0.047	n = 12, n = 11	138	0.0090	FIR
15	− 0.030	n = 15, n = 14	277	0.0045	FIR
20	− 0.017	n = 20, n = 19	675	0.0018	FIR

5 $${\Delta E}_{HFS}=\frac{203.4}{{n}^{3}}$$

The results for Eq. (5) are listed in Table 2. All ΔE_HFS_ lie in microwave range, hence inverse shielding for a PET-MR hybrid unit is to block external microwave sources, most likely from 0.025GHz (n = 20) to 203.4GHz (n = 1). For instance, the frequencies for analog TVs are 0.05GHz to 0.9GHz; the mobile phone frequencies in the US are 1 ~ 3GHz; and the frequency for a microwave oven is ~ 12GHz. Thus, the HFS spectrum covers a broad spectrum of almost all electronic devices using microwaves.

**Table 2:** Positronium HFS energy for different principal quantum numbers n.

Quantum number	Energy states	HFS (GHz)	Spectrum
1	1^3^S_1_, 1^1^S_0_	203.4	EHF (MV): NATO N band, satellite
2	2^3^S_1_, 2^1^S_0_	25.42	SHF (MV): radio astronomy, wireless LAN, most radar, Satellite communication, satellite phone (S band)
3	3^3^S_1_, 3^1^S_0_	7.533	
4	4^3^S_1_, 4^1^S_0_	3.178	
5	5^3^S_1_, 5^1^S_0_	1.627	UHF: MW oven, TV, Bluetooth radar (L band), cable TV, GPS, mobile phone, cardless phone, satellite broadcast, internet, police
6	6^3^S_1_, 6^1^S_0_	0.9416	
7	7^3^S_1_, 7^1^S_0_	0.5930	
8	8^3^S_1_, 8^1^S_0_	0.3972	
9	9^3^S_1_, 9^1^S_0_	0.2790	VHF: MRI, FM radio, TV, radar, cable TV, cardless phone, ground-to-aircraft and aircraft-to-aircraft communication, police
10	10^3^S_1_, 10^1^S_0_	0.2034	
12	12^3^S_1_, 12^1^S_0_	0.1177	
15	15^3^S_1_, 15^1^S_0_	0.06026	
20	20^3^S_1_, 20^1^S_0_	0.02542	HF: MRI, cardless phone

### Options on inverse shielding

It seems candid to make a rule to forbid using electronic devices of microwave spectrum inside the PET-MR hybrid vault. However, in a typical MR scanner operation manual, there should be restrictions for those electronic devices inside the MR examination room to avoid interferences to MR imaging, which are also applicable to PET-MR hybrid unit to ensure PET imaging quality. Table 2 indicates that HFS spectra overlap with MR radiations at low frequencies (n ≥ 9), although HFS of higher frequencies (n ≤ 8) lie in microwave bands. Since the MR restrictions are typically for all electronic devices, forbidding all bandwidths, they are redundant for MR imaging, but also suitable for PET imaging quality protection.

The ideal shielding material for external microwave outside the vault is a metal layer—even a very thin metal layer, e.g., aluminum layer of 1mm, could absorb those microwaves 100%. For this reason, a metal layer (aluminum, copper, steel, or lead) should be always put into PET-MR vault shielding at all directions. However, most MR examination rooms are already shielded redundantly with metallic *Faraday Cage*, to avoid external radiations entering the MR unit. The *Faraday Cage* might also be significant for microwave shielding, even though its original focus was on shielding external radio waves which could potentially interfere MR imaging. For this reason, a *Faraday Cage* could be kept for a PET-MR hybrid unit, but it should be carefully examined, and possibly amended if necessary, because its original purpose was for MR unit alone, whereas microwaves are more penetrating with much shorter wavelengths.

### Mutually exclusive working mode for PET and MR imaging

The MR imaging requires excitation pluses of frequencies close to the Lamour frequency of the nuclei (e.g., protons), which are generally from 1 to 300MHz, and those radio signals overlap with the HFS energies (Table 2, n ≥ 9)^41^. The excitation pulses always penetrate the body, where radionuclide e.g., FDG, was already injected, hence the 3γ/2γ ratio will inevitably increase and degrade PET contrast resolution. The only solution seems to be vetoing each other automatically during the imaging. That is, create a mutually exclusive work mode for the hybrid unit, such that there are no mutual interferences. While MR is collecting data, the PET is not, and vice versa. However, there are 2 factors to be considered. First, the HFS overlap with MR frequency at higher excited energy levels (n ≥ 9), whereas lower energy levels are always much more populated. Second, the MR radiations are not continuous in spectrum. For those 2 reasons, the chance that HFS frequencies match MR radiations for interference is quite small, hence mutual interference of PET and MR might be insignificant in hybrid imaging.

## Conclusions

For the first time, we conducted research on PET imaging quality of PET-MR hybrid scanning based on positronium HFS transitions under strong MR magnetic field. Prevalent PET imaging only collects 2γ events annihilated from p-Ps and ignores 3γ annihilations from o-Ps as noises, thus the 3γ/2γ ratio is crucial for clinical PET imaging quality. If no other factors are introduced the 3γ/2γ ratio is ~ 3/7 in biological tissues. Due to *pick-off quenching* for o-Ps, many o-Ps converts to p-Ps and ultimately decay to 2γ, reducing the 3γ/2γ ratio hence improving the contrast resolution of PET. In addition, known as *Magnetic Quenching* in PET-MR hybrid imaging, the MR magnetic field conducts transition pathways between some split states of o-Ps and p-Ps, many o-Ps could spontaneously convert to p-Ps by emitting HFS photons, since a p-Ps is at lower energy state for the same quantum numbers. This could also improve PET imaging quality automatically. However, *Inverse Magnetic Quenching* for p-Ps due to induce HFS excitations might also occur if radio wave sources of HFS frequencies are available nearby, that is, many p-Ps could convert to o-Ps by absorbing HFS photons if sources of HFS are available, hence significantly degrades the PET contrast. The HFS spectrum covers a broad range of electromagnetic waves, from 0.02-200GHz. For this reason, electronic devices lie in those bands inside are forbidden in PET-MR hybrid unit vaults. However, most MR operation manual includes similar restrictions hence could be adopted for PET-MR hybrid examinations. On the other hands, inverse shielding against external microwave sources is necessary for PET-MR vault. However, most MR units were already redundantly shielded with metallic *Faraday Cage* against external radio waves. This *Faraday Cage* could be also significant for microwave shielding although its original purpose is for MR alone, however, it should be carefully examined, probably amened, to fit the requirement of PET-MR hybrid imaging. The radio frequencies generated by MR unit partially overlap with HFS regime of high energy levels, however, the chance for mutual interference is so small that mutually exclusive working mode might not be needed for most of situations.

## Data Availability

The datasets generated in this study are in simple formats and are available from the corresponding author per reasonable requests.

## References

[1] Charron M, Beyer T, Bohnen NN, Kinahan PE, Dachille M, Jern J, Nutt R, Meltzer CC, Villemagne V, Townsend DW (2000). Image analysis in patient with cancer studied with a combined PET and CT scanner. Clin. Nucl. Med..

[2] Beyer T, Townsend DW, Brun T, Kinahan PE, Charron M, Roddy R, Jerin J, Young J, Byars L, Nutt R (2000). A combined PET/CT scanner for clinical oncology. J. Nucl. Med..

[3] Pichler BJ, Judenhofer MS, Wehrl HF (2008). PET/MR hybrid imaging: Devices and initial results. Eur. Radiol..

[4] Antoch G, Bockisch A (2009). Combined PET/MRI: A new dimension in whole-body oncology imaging. Eur. J. Nucl. Med. Mole Imaging.

[5] Razifar P, Sandstorm M, Schnieder H, Langstrom B, Maripuu E, Bengtsson E, Bergstrom M (2005). Noise correction in PET, CT, SPECT and PET/CT data evaluated using autocorrection function: A phantom study on data, reconstructed using FBP and OSEM. BMC Med. Imaging.

[6] Olcott PD, Peng H, Levin CS (2009). Novel electro-optical coupling technique for magnetic resonance-compatible positron emission tomography detectors. Mol. Imaging.

[7] Vandenberghe S, Marsden PK (2015). PET-MRI: A review of challenges and solutions in the development of integrated multimodality imaging. Phys. Med. Biol..

[8] Yoo HJ, Lee JS, Lee JM (2015). Integrated whole body MR/PET: Where are we?. Korean J. Radiol..

[9] Herzog H, Van Den Hoff J (2012). Combined PET/MR systems: An overview and comparison of currently available options. Q J. Nucl. Med. Mol. Imaging.

[10] Judenhofer MS, Catana C, Swann BK, Siegel SB, Jung WI (2007). PET/MR images acquired with a compact MR-compatible PET detector in a 7-T magnet. Radiology.

[11] Keereman V, Mollet P, Berker Y, Schulz V, Vandenberghe S (2013). Challenges and current methods for attenuation correction in PET/MR. Magn. Reson. Mater. Phys..

[12] Ouyang J, Li Q, Fakhri E (2013). Magnetic resonance-based on motion correction for positron emission tomography imaging. Semin. Nucl. Med..

[13] Gravel P, Verhaeghe J, Reader AJ (2013). 3D PET image reconstruction including both motion correction and registration directly into an MR or stereotaxic spatial atlas. Phys. Med. Biol..

[14] Marin T, Djebra Y, Han PK, Chemli Y, Bloch I, Fakhri GE, Ouyang J, Petibon Y, Ma C (2020). Motion correction for PET data using subspace-based real-time MR imaging in simultaneous PET/MR. Phys. Med. Biol..

[15] Fürst S, Grimm R, Hong I, Souvatzoglou M, Casey ME, Schwaiger M, Nekolla SG, Ziegler SI (2015). Motion correction strategies for integrated PET/MR. Nucl. Med..

[16] Garwin RL (1953). Thermalization of positrons in metals. Phys. Rev..

[17] Mackintosh, A. R. Positron annihilation in solids, by Generic, 1962 (Reprint in 2018).

[18] Hautojervi, P. *Positrons in Solids* (Springer Verlag, 1979). ISBN-13, 978–0387092713.

[19] Moskal P, Jasinska B, Stepien EL, Bass SD (2019). Positronium in medicine and biology. Nat. Rev. Phys..

[20] Vetter PA, Freedman SJ (2002). Branching-ratio measurements of multiphoton decays of positronium. Phys. Rev. A.

[21] Govaerts J, van Caillie M (1996). Neutrino decay of positronium in the Standard Model and beyond. Phys. Lett. B.

[22] Yamamato S, Higashi T, Matsumoto K, Senda M (2006). Development of positron-imaging detector with background rejection capability. Ann. Nuclear Med..

[23] Bergeron M, Cadorette J, Tetrault M-A, Beaudoin J-F, Leroux J-D, Fontaine R, Lecomte R (2014). Imaging performance of LabPET APD-based digital PET scanners for pre-clinical research. Phys. Mod. Biol..

[24] Ollinger JM (1995). Detector efficiency and Compton scatter in fully 3D PET. IEEE Trans. Nucl. Sci..

[25] Cherry SR, Joes T, Karp JS (2018). Total-body PET: Maximizing sensitivity to create new opportunities for clinical research and patient care. J. Nucl. Med..

[26] Ore A, Powell JL (1963). Three-photon annihilation of an electron-positron pair. Phys. Rev..

[27] Heiss MW, Wichmann G, Rubbia A, Crivelli P (2018). The positronium hyperfine structure: Progress towards a direct measurement of the 2^3^S_1_ → 2^1^S_0_ transition in vacuum. J. Phys..

[28] Karshenboim SG (2004). Precision study of positronium: Testing bound state QED theory. Int. J. Mod. Phys. A..

[29] Moskal P, Kisielewska D, Shopa RY, Bura Z, Chhokar J (2020). Performance assessments of 2γ positronium imaging with the total-body PET scanner. EJNMMI Phys..

[30] Jasinska B, Moskal P (2017). A new PET diagnostic indicator based on the ratio of 3γ/2γ positron annihilation. Acta Phys. Polon. Ser. B.

[31] Gladen, R. W., Chirayath, V. A., & Fairchild, A. J., *et al.* Estimation of ortho-positronium pick-off contributions to coincidence measurement of the energy spectrum of positron-induced electrons and annihilation γ quanta. LLNL-PROC-812543 (2020).

[32] Siegel RW (1980). Positron annihilation spectroscopy. Annu. Rev. Mater. Sci..

[33] Corbel C, Stucky M, Hautojärvi P, Saarinen K, Moser P (1988). Positron-annihilation spectroscopy of native vacancies in as-grown GaAs. Phys. Rev. B Condens. Matter.

[34] Dlubek G, Pionteck J, Bondarenko V (2002). Positron annihilation lifetime spectroscopy (PALS) for interdiffusion studies in disperse blends of compatible polymers: A quantitative analysis. Macromolecules.

[35] Jasinska B, Zgardzinska B, Cholubek G (2017). Human tissue investigations using PALS technique—free radicals influence. Acta Phys. Polon. A.

[36] Heremans K (2005). Protein dynamics: Hydration and cavities. Braz. J. Med. Biol. Res..

[37] Han X, Gao J, Chen T, Qian L, Xiong H, Chen Z (2022). Application progress of PALS in the correlation of structural and properties for graphene/polymer nanocomposites. Nanomaterials (Basel).

[38] Halpern O (1954). Magnetic quenching of the positronium decay. Phys. Rev..

[39] Consolati G (1996). Magnetic quenching of positronium. J. Radioanal. Nucl. Chem. Articles.

[40] Liu JD, Guo JQ, Luo M, Wang Z, Zhang HJ, Ye BJ, Chen ZQ (2020). Magnetic quenching of positronium studied by positron annihilation lifetime and Doppler broadening measurements. Rad. Phys. Chem..

[41] Niesporek SC, Nagel AM, Platt P (2019). Multinuclear MRI at ultrahigh fields. Top. Magn. Reson. Imaging.

